# The Use of Dissimilar Metals in Filling a Tooth

**Published:** 1878-06

**Authors:** A. W. Harlan

**Affiliations:** Chicago, Ill.


### ARTICLE V.

### *Concerning the Use of Dissimilar Metals in Filling a Tooth.*

BY A. W. HARLAN, CHICAGO, ILL.

Since it is the fashion now-a-days for everyone, be he large or small, to have his ideas concerning the practice of dentistry aired in some publication, I trust that the readers of THE JOURNAL will allow me to offer them something which has not, heretofore, been much discussed; indeed I have been able to find but the slightest reference to it in the text-books on dentistry, and a pretty thorough search of the transactions of societies and periodical literature has disclosed nothing very valuable or scientific concerning what is to follow:

I have long entertained the opinion that it was not good practice to use gold and amalgam in the same tooth, whether the fillings were seperated from each other or not. Observation has shown me that a great many dentists are in the habit of filling teeth in the above manner, and I have been endeavoring to collect a sufficient number of good reasons to convince them that it is not a very wise method of operating. I will first cite cases from my own practice, and then give my experiments on the subject.

March, 1870, filled large crown cavity and roots of lower first molar with amalgam; filled buccal cavity in the same tooth with gold; the metals did not come in contact. March, 1878, eight years afterwards, the buccal filling had to be renewed. Crown filling in good condition yet. May, 1870, filled upper second molar in the same mouth, distal cavity including roots, using amalgam; filled mesial cavity with gold. February, 1878, was compelled to refill the mesial cavity on account of the recurrence of decay around the filling. July, 1877, filled buccal cavity in lower first molar (alive) with amalgam, and crown cavity with gold. August, 1873, crown filling had to be replaced in order to save the tooth. August, 1871, filled distal cavity in upper second bicuspid (alive) with amalgam, and mesial cavity in the same tooth with gold. September, 1877, renewed the mesial filling for the same reason that the others were refilled.

In the few cases where I have built gold up by the side of an amalgam filling I have found that as a rule *decay is likely to recur around the margins of both fillings equally*, and also where I have used amalgam to fill a cavity by the side of a gold the result was the same.

I ceased several years ago using two metals in one tooth, whether alive or devitalized, because I was convinced that one of the cavities would sooner or later have to be refilled. I was as much surprised to find that decay almost invariably attacked the margins around the gold filling, as any one of the readers of THE JOURNAL can be at my recording it, but such is the result of my observations, regarding my own operations and those from the hands of other reputable practitioners.

Now for the experiments: Selecting a perfectly sound tooth I filled the crown with amalgam and the buccal surface with gold, and suspended the crown in a solution of cider vinegar of one-tenth strength, allowing it to remain 48 hours. On removal it was very easy to scrape off the enamel around the gold filling, and an almost imperceptible amount of softening had taken place around the amalgam filling. Repeated the same experiment with a one-twentieth solution of vinegar, allowing the tooth to remain seven days with the same result precisely. Experimented once more, using this time a central incisor, the proximal surfaces of which were filled—one with amalgam and the other with gold, allowing it to remain suspended eight days in a one-twenty fifth solution, with no different result.

If these experiments and practical cases prove anything, it is that *we should not expect to save a tooth by using two metals in it, for the purpose of arresting caries.* We must not for the sake of economy or of shortening an operation fill an immense cavity with amalgam and a small one with gold, but on the other hand, it would seem imperative that the dentist should fill both cavities with one material, whether it be of amalgam or of gold. I have been practising in this way since 1872, and have not, and will not fill a tooth unless I can use one metal for filling all the cavities in it. Without being sure of it, I may say that I believe no bad results need be apprehended from using tin and amalgam in the same tooth, or tin and gutta-percha or oxychloride of zinc, etc, but I do not use gold and tin in the same tooth when they are seperated from each other by a portion of tooth substance, because the tin, if not worn out by mastication, is dissolved by the acid secretions of the mouth. I do not pretend to explain the reason why decay recurs around the margins of a gold filling when there is an amalgamfillinginthe same tooth, but will leave that to the experimenter with electricity.

With reference to the use of tin and gold in combination for filling teeth, I am of the opinion that if either of these metals are used honestly and thoroughly the result will be the same, that is to say, the tooth will be saved and its color will be better if the metals are used separately. I venture to hint that after the lapse of sufficient time—say three years, the combination fillings of tin and gold will begin to flake and become depressed on the grinding surface of the teeth, and the proximal surfaces turn quite black, which in both cases would be calamitous.

In conclusion it will not be presumptuous, I hope, to remark that the dentist who wishes to serve his patients best, will use his own judgment in the selection of materials for filling, and his own method for accomplishing the desired end, and at the same time not permit pecuniary considerations to come between him and the salvation of a tooth, remembering that it is better to save one tooth for useful service, than to miserably fail in saving a dozen teeth by following the advice or wishes of a patient, thereby confessing your want of firmness and want of judgment.—*Missouri Dental Journal.*

